# Age-dependent changes of hindgut microbiota succession and metabolic function of Mongolian cattle in the semi-arid rangelands

**DOI:** 10.3389/fmicb.2022.957341

**Published:** 2022-07-22

**Authors:** Zeyi Liang, Jianbo Zhang, Mei Du, Anum Ali Ahmad, Shengyi Wang, Juanshan Zheng, Ghasem Hosseini Salekdeh, Ping Yan, Jianlin Han, Bin Tong, Xuezhi Ding

**Affiliations:** ^1^Lanzhou Institute of Husbandry and Pharmaceutical Sciences, Chinese Academy of Agricultural Sciences, Lanzhou, China; ^2^Agricultural Biotechnology Research Institute of Iran, Agricultural Research, Education, and Extension Organization, Karaj, Iran; ^3^International Livestock Research Institute (ILRI), Nairobi, Kenya; ^4^School of Life Sciences, Inner Mongolia University, Hohhot, China

**Keywords:** Mongolian cattle, hindgut, microbiota, colonization, metabolome

## Abstract

Dietary changes have significant effects on gut microbiota and host health. Weaning is an important stage of dietary change in ruminants. The gastrointestinal tract (GIT) microbiota of calf in the early life undergo some changes, and the plasticity of the calf is beneficial to cope with these changes and challenges. However, the complex development of hindgut microorganisms in post-weaning ruminants is not fully understood. In this study, we used 16S rRNA sequencing and untargeted metabolomic analysis to determine the cecal and colonic bacterial community and associated metabolome of Mongolian cattle at age of the 5th (at weaning), 18th, and 36th months. Moreover, the maturation patterns of the hindgut bacterial community and the dynamic changes of metabolites were also explored. Sequencing results showed that Firmicutes and Bacteroidetes were the dominant phyla in the cecum and colon. The linear discriminant analysis (LDA) effect size (LEfSe) analysis revealed bacterial features that were stage-specific in the cecum and colon. The relative abundance of Ruminococcaceae, a microbial family related to fiber degradation, gradually increased with age in the cecum, while the relative abundance of *Bacteroides* and *Alistipes*, which are related to immunity, gradually increased in the colon. The differential metabolites in the cecum and colon were mainly enriched in steroid hormone biosynthesis, primary bile acid biosynthesis, and arachidonic acid metabolism between different ages of Mongolian cattle after weaning. Consequently, this dual omics analysis provided important information on the changes in microbial and metabolite interactions in Mongolian cattle after weaning. The microorganisms and metabolites in the cecum and colon further enhanced the abiotic stress resistance of Mongolian cattle to the harsh environment. The information obtained in this study is of great significance for future strategies of cecum and colon microbiota regulation of post-weaning Mongolian cattle in the harsh Mongolian Plateau ecosystem.

## Introduction

The gastrointestinal tract (GIT) inhabiting trillions of microorganisms is the most complicated and largest microecosystem playing vital roles in the host metabolism, growth performance, and health of various animals (Xiong, 2022; Yan et al., 2022; Yu et al., 2022; Zhu et al., 2022). The microorganisms colonized in the small and large intestines are considered to be essential for the energy-gathering function and health of the ruminants (Falcinelli et al., 2015; Leng, 2018; Lopes et al., 2019). Recent research found higher clustering coefficients of co-correlated metagenomic assembly genome (MAG) in the large intestine of the ruminants (Xie et al., 2021), thus suggesting the importance of microbes in the large intestine.

Early gut microbiome establishment is associated with health and growth (neonatal diarrhea, feces, and weight gain) of the calf, and recent studies have found that weaning has a greater impact on the development of gastrointestinal microbiota of calves than weaning strategies (Zhang et al., 2021). The parent–child relationship of the ruminants reaches a new stage after the weaning of the young ruminants (Enríquez et al., 2011), and the diet of the ruminants changes from milk-derived fats and amino acids to forage or feed with a greater amount of fiber. The newly established social groups among the animals and the rapidly emerging external environments may cause psychological, physiological, and immune response challenges to the young animals (O’Loughlin et al., 2014). Therefore, it is of great importance to study the GIT microbial composition, metabolites, and their correlation to a better understanding of host-microbe interaction. At present, few studies have characterized the stressful transition of the gut microbiome from post-weaning to mature ruminants, so the effects of the post-weaning on the intestinal microbiome on host adaptation are largely unknown.

Mongolian cattle, one of the East Asian indigenous Bos taurus (taurine cattle), is mainly distributed in the Inner Mongolia Autonomous Region of China, Mongolia, and the East Siberian Region of Russia (Chen et al., 2021). The Mongolian region is centered by the Mongolian Plateau with a temperate continental climate characterized by drought, high altitude, and large differences in day and night temperatures. Mongolian cattle can adapt to a low-quality diet to withstand harsh conditions. They can also digest almost any plant, such as Gramineae, Compositae, and Leguminosae plants. Gut microbiota plays important roles in host animal metabolism, homeostasis, and environmental adaptation (Wei et al., 2020). While most previous studies focused on the development of rumen function and microbial succession (Li R. W. et al., 2012; Rey et al., 2012; Jiao et al., 2015b), the trajectory of hindgut microbiota remains to be uncovered. Therefore, the purpose of this study was to compare the community structure and composition of the colonic and cecal microbiota of Mongolian cattle at different ages after weaning and to reveal the phylogenetic composition, maturation, and interaction of the microbiota in different parts of the hindgut of Mongolian cattle, to provide scientific basis for the research and healthy breeding of Mongolian cattle in the future.

## Materials and methods

### Animals and sample collection

All procedures involving animal care and use were in strict accordance with the Guide for the Care and Use of Laboratory Animals, Lanzhou Institute of Husbandry and Pharmaceutical Sciences, CAAS, China [SCXK (Gan) 2014- 0002].

The Mongolian cattle grazed naturally year-round on native pastures from Alxa Left Banner of Alxa League, Inner Mongolia Autonomous Region (105.68 E, 38.85 N; altitude 1,522 m). The region belongs to a typical continental climate, characterized by scarce precipitation, intense sunshine, great changes in cold and heat, and unusual drought. Fifteen Ujumqin Mongolian cattle at the age of the 5th, 18th, and 36th months were randomly selected from a farm in Alxa Left Banner. Animals were selected for the same feeding regimen, in which all animals were weaned naturally at the 5th month of age and exclusively breastfed until weaned. At the age of the 5th month, the Mongolian calves were weaned and allowed to accompany their mothers to the pasture for grazing. After weaning, animals are allowed to graze on natural alpine shrubby grasslands all year round and drink water from local rivers. Vegetation in this area is dominated by *Salix cupularis*, *Haloxylon ammodendron*, *Caragana jubata*, and *Kobresia* spp., and these plants have high fiber content. The sampling time in this study was in winter when the Mongolian Plateau was dry and cold and forage resources were scarce. None of the studied animals had been pregnant or given birth before. The animals used in the study were also not treated with any antibiotics. Moreover, animals were not provided with any supplementary concentrate feed before or after weaning or later in life. Therefore, we selected Mongolian cattle each during the after-weaning (approximately the 5th month of age), growing period (approximately the 18th month of age), and mature period (approximately the 36th month of age), with five cattle in each period. Fifteen Mongolian cattle were slaughtered in December 2020, and fresh samples of colon and cecum contents were obtained. To minimize the possible mixture of contents across GIT regions, different parts of the gut were tied off using cotton rope after the animals were slaughtered by traditional Mongolian butchers. The colon and cecum contents were collected, and snap-frozen in liquid nitrogen before storage at −80°C for further analysis.

### Genomic DNA extraction and high-throughput sequencing

Total genomic DNA was extracted from luminal contents of colon and cecum contents using the DNeasy PowerSoil Kit (Cat. No. 12888, Qiagen, Hilden, Germany) according to the manufacturer’s protocols. The concentration and quality of DNA were assessed using a NanoDrop 2000 UV-Vis Spectrophotometer (Thermo Scientific, Wilmington, DE, United States) and agarose gel (1.0%) electrophoresis, respectively. The isolated DNA was stored at −20°C until downstream analysis. For cecum and colon contents microbial profiling, primers 343 F 5′-TACGGRAGGCAGCAG-3′ and 798 R 5′-AGGGTATCTAATCCT-3′ targeting the V3 and V4 hypervariable regions of the bacterial partial 16S rRNA gene were used to generate amplicons for cecum and colon microbiota (Wang et al., 2021). The PCR amplification products were verified using agarose gel (2%) electrophoresis and purified with the QIAamp 96 PowerFecal QIAcube HT Kit (51331 QIAGEN, Valencia, CA, United States). After the second purification with the AMPure XP beads, the final amplicon was quantified using the Qubit™ dsDNA HS Assay Kit (Q32854, Thermo Fisher Scientific).

High-throughput sequencing of amplicons was performed using the Illumina Mi-Seq platform (Illumina, San Diego, CA, United States). Raw FASTQ files were quality-filtered by Trimmomatic v0.33 (Bolger et al., 2014) and merged by FLASH v1.2.112 (Butler et al., 2003) according to the following criteria: the low-quality sequences were cut off with an average quality score below 20 using a sliding window trimming approach. Parameters of assembly were: 10 bp of minimal overlapping, 200 bp of maximum overlapping, and 20% of maximum mismatch rate. Sequences were treated further for denoising as follows: reads with ambiguous and homologous sequences or below 200 bp were abandoned. Reads with 75% of bases above Q20 were retained. Then, reads with chimera were detected and removed. These two steps were achieved using QIIME 2 software version 2020.6 with the DADA2 pipeline (Ishizaka et al., 2021). Clean reads were subjected to primer sequences removal and clustering to generate amplicon sequence variants (ASVs) using VSEARCH software (Rognes et al., 2016) with a 100% similarity cutoff. The representative read of each ASV was selected using QIIME software. All representative reads were annotated and blasted against SILVA database version 132 using RDP Classifier version 2.2 (Wang et al., 2007) (confidence threshold was 70%) and also the UNITE v7 database (Altschul et al., 1990).

### Sequencing data analysis

For alpha diversity, all analyses were based on the ASV clusters with a cutoff of 3% dissimilarity. Chao1, Shannon, Simpson, and Faith’s PD were calculated to estimate the richness and diversity of the bacterial community of each sample three times (the 5th, 18th, and 36th months of age) separately. Rarefaction curves based on the average number of observed ASVs were generated using the Mothur software package. For β-diversity, Bray–Curtis distance metrics were used to generate PCoA to further assess the similarities between the community compositions of different samples, and one-way PERMANOVA (followed by pairwise PERMANOVA tests when the one-way tests resulted in *P* < 0.05) was used to analyze the differences between groups. Heatmaps and Venn diagrams were generated using “VennDiagram” (v 1.6.20) and “pheatmap” (v 1.0.12) package within R version 3.4.0 (Wiener et al., 2003; Bouchareychas et al., 2020). Histograms were created in Microsoft Excel 2019.

### Profiling the metabolites of colonic and cecal contents

A total of 30 samples of cecal and colonic contents were analyzed using the Dionex™ UltiMate™ 3000 Biocompatible Rapid Separation (BioRS) system fitted with Q Exactive™ Plus Hybrid Quadrupole-Orbitrap™ Mass Spectrometer equipped with heated electrospray ionization (ESI) source (Thermo Fisher Scientific, Waltham, MA, United States) for their metabolic profiling in both ESI positive and negative ion modes. All the samples were kept at 4°C during the analysis. Data acquisition was performed in full scan mode (m/z ranges from 70 to 1000) combined with IDA mode. The raw data were analyzed by the progenesis QI software (Waters Corporation, Milford, MA, United States) using the following parameters. The precursor tolerance was set at 5 ppm, fragment tolerance at 10 ppm, and retention time (RT) tolerance at 0.02 min. Internal standard detection parameters were deselected for peak RT alignment, isotopic peaks were excluded for analysis while noise elimination level was set at 10.00 and minimum intensity at 15% of base peak intensity. The internal standard was used for data QC. Metabolites were identified by progenesis QI based on public databases such as Human Metabolome Database,^1^ Lipid Metabolites, and Pathways Strategy^2^ and a self-built database. Principle component analysis (PCA) and (orthogonal) partial least-squares-discriminant analysis (PLS-DA) was carried out to visualize the metabolic alterations among experimental groups after mean centering (Ctr) and Pareto variance (Par) scaling, respectively. The differential metabolites were selected based on the combination of a statistically significant threshold of variable influence on projection (VIP) values obtained from the PLS-DA model and *P*-values from a two-tailed Student’s test on the normalized peak areas, where metabolites with VIP values > 1.0 and *P*-values < 0.05 were considered as differential metabolites. Moreover, the concentrations of volatile fatty acids (VFAs) in hindgut contents were determined according to the method of a previous study (Jiao et al., 2015a). In brief, 5 mL cecal or colonic content was dissolved in 1 mL 25% metaphoric acid (wt/vol) containing 2 g/L internal standard 2EB (2-ethylbutyric acid) and then centrifuged at 15,000 × *g* for 15 min at 4°C. The gas chromatography (Agilent Technologies 6890 GC, Santa Clara, CA, United States) with a DB-FFAP column (30 μm × 0.25 μm × 0.25 μm) was applied to measure the concentrations of volatile fatty acids (VFAs) in the supernatant.

### Correlation analysis

Correlation analysis between microbiomes and metabolites in cecum and colon was performed using Spearman’s rank correlation in SPSS v25.0 to identify the microbial community succession associated with types of metabolites, with *P*-value (Spearman’s rank correlation coefficient) <0.05 being considered significant. To determine the relationship between microbial composition and metabolite covariate, we performed the analysis of variance (ANOVA) on the microbial abundance profiles using microbial Bray–Curtis distance using the R “vegan” package (v 2.5-7) within the R version (3.4.0.) (Siranosian et al., 2022). Metabolites with FDR-adjusted *P* < 0.05 were associated with microbiota. The nodes in the network represent cecum and colon taxa and differential metabolites. All correlation analyses were performed using Spearman’s rank correlation, and *P*-value < 0.05 was considered significant. The correlation network was visualized by Cytoscape version 3.7.1.^3^ Other topological features, such as the degree centrality, transitivity, and closeness centrality, were also calculated using the “igraph” package version 1.2.6^4^ to describe the complexity of this network. Nodes with the highest betweenness centrality scores were keystone species in the co-occurrence networks.

## Results

### Summary of 16S rRNA sequencing and alpha diversity

A total of 1,353,348 and 1,346,858 high-quality 16S rRNA gene sequences were generated from the cecum and colon samples across the three age groups, respectively. The average numbers of sequences for the cecum and colon samples were 90,233 and 89,709, respectively. Based on 100% of sequence similarity, up to 2,428 and 2,317 bacterial ASVs were identified for the cecum and colon, respectively. Rarefaction curves showed a diminishing rate of new ASVs identification as the number of reads per sample increased, implying that the sequencing depth was adequate for evaluating the dominant bacterial community (Supplementary Figures 1, 2).

The distribution of bacterial ASV in the cecum showed 16 shared ASV between animals at the 5th and 36th months (accounting for 0.65% of the total ASV), 1,197 shared ASV between animals at the 18th and 36th months (accounting for 49.34% of the total ASV), and finally, only 135 shared ASV were identified between animals at the 5th and 18th months (accounting for 5.56% of the total ASV) (Supplementary Figure 3A). In conclusion, the plasticity of cecal microorganisms after weaning and with age change of Mongolian cattle further promote the gradual maturation of the microbial community. The distribution of bacterial ASV in the colon revealed 551 shared ASV, and Mongolian cattle colon microbes had more shared flora in three age groups (about 22.01% of total ASV) (Supplementary Figure 3B).

### Profile and characteristics of the cecum and colon microbial community of Mongolian cattle at the 5th, 18th, and 36th months of age

**FIGURE 1:**
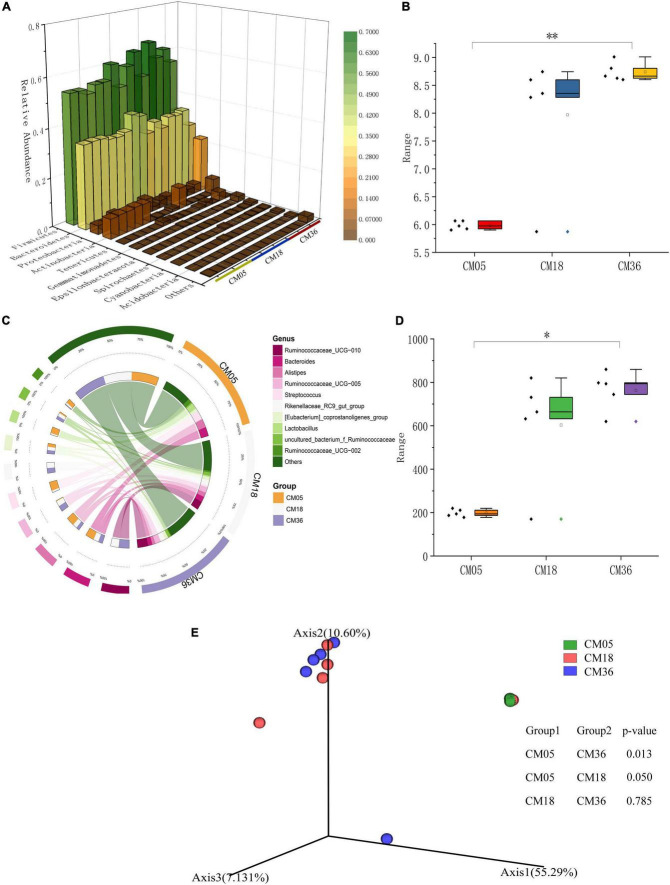
Relative abundance and diversity of bacterial communities in the cecum of post-weaning Mongolian cattle. **(A)** Taxonomic composition of the bacterial phylum, red indicates high relative abundance and green indicates low relative abundance. **(B)** Shannon index of bacteria in each group (**P < 0.01). **(C)** Taxonomic composition of the bacterial genera. Groups are shown on the left side of the large circle, the relative abundance of bacteria is shown on the left side, and the percentage of bacteria in each group is shown on the small circle. **(D)** Chao1 index of bacteria in each group (*P < 0.05). **(E)** Principal co-ordinate analysis of cecal bacteria in post-weaning Mongolian cattle.

To gain insight into the diversity of the cecal bacterial community, we compared the Chao1 and Shannon indices across the three age groups. In cecal samples, the diversity and richness of microorganisms increased significantly with the maturity of the host (Figures 1B,D and Supplementary Tables 1, 2). The principal co-ordinate analysis (PCoA) showed clear age-based separation of cecal bacteria between the 5th and 36th months, Axis 1 explaining 55.29% of the total variation (Figure 1E).

GIT bacterial community composition was analyzed at the phylum and genera levels. In total, 29 bacterial phylum were detected (average relative abundance >0.1%) in the cecum with Firmicutes and Bacteroidetes being predominant, accounting for around 90% of the total bacterial taxa identified at the age of the 5th (53.36 and 34.05%), 18th (57.25 and 35.78%), and 36th (55.30 and 32.72%) months (Figure 1A and Supplementary Table 3). In the 5th month, the *Bacteroides* (12.68%) was the predominant, followed by the genera *Streptococcus* (12.24%), *Alistipes* (12.10%), and *Lactobacillus* (8.88%), which altogether made up 45.90% of the bacterial composition (Figure 1C and Supplementary Table 4). In the 18th month, microorganisms associated with fiber degradation increased in the cecum, the *Ruminococcaceae UCG-010* (11.99%) was the most common, followed by the genera *Bacteroides* (7.69%), *Ruminococcaceae UCG-005* (7.65%), *Rikenellaceae RC9 gut group* (7.57%), *Alistipes* (6.38%), and *[Eubacterium] coprostanoligenes group* (4.76%), all accounting for up to 46.04% of the bacterial community. The dominant microorganisms in cecum at the 36th month of age were almost the same as those at the 18th month of age, the *Ruminococcaceae UCG-010* (15.80%) was the most abundant, followed by the genera *Ruminococcaceae UCG-005* (8.42%), *Rikenellaceae RC9 gut group* (7.06%), *Bacteroides* (6.32%), and *[Eubacterium] coprostanoligenes group* (4.90%), all of them accounting for 42.91% of the bacterial taxa.

**FIGURE 2:**
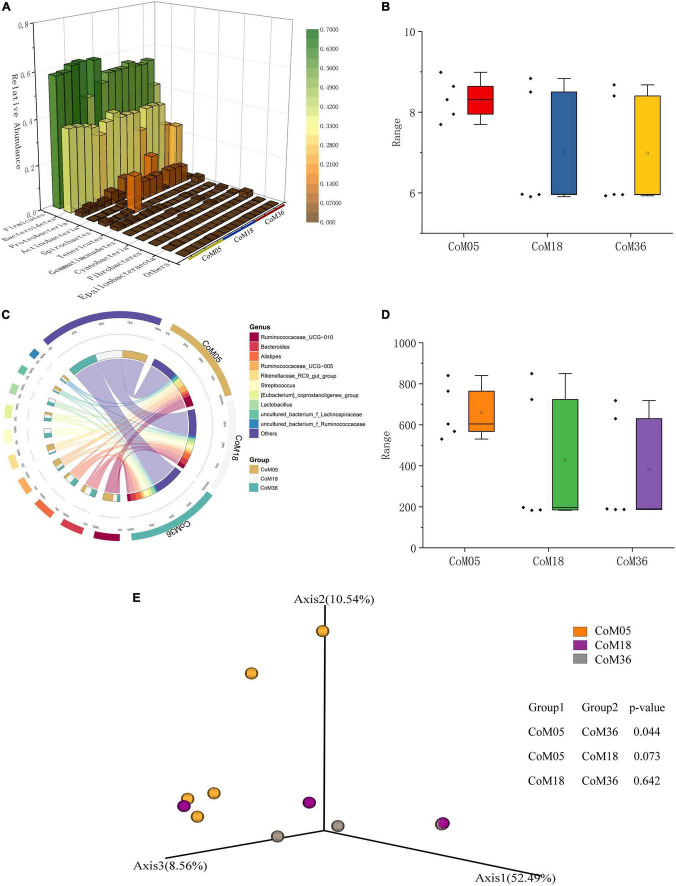
Relative abundance and diversity of bacterial communities in the colon of post-weaning Mongolian cattle. **(A)** Taxonomic composition of bacterial phylum, red indicates high relative abundance and green indicates low relative resolution. **(B)** Shannon index of bacteria in each group. **(C)** Taxonomic composition of the bacterial genera. Groups are shown on the left side of the large circle, the relative abundance of bacteria is shown on the left side, and the percentage of bacteria in each group is shown on the small circle. **(D)** Chao1 index of bacteria in each group. **(E)** Principal co-ordinate analysis of colon bacteria in post-weaning Mongolian cattle.

To gain insight into the diversity of the colonic bacterial community, we compared the Chao1 and Shannon indices across the three age groups. There was no significant difference in the Chao1 and Shannon in the colonic bacterial community across the three age groups (*P* > 0.05) (Figures 2B,D and Supplementary Tables 1, 2). The PCoA plot showed a clear age-based separation of colonic bacteria in the Axis 2 direction between the 5th and 36th months (Figure 2E).

A total of 15 bacterial phyla were identified from the colonic bacteria of Mongolian cattle across the three age groups. At the phylum level, both Firmicutes and Bacteroidetes were most common across the three age groups, accounting for 91.11, 89.28, and 80.35% of all bacterial taxa in the colon at the 5th (56.75 and 34.47%), 18th (54.32 and 35.18%), and 36th (51.73 and 30.70%) months of age, respectively (Figure 2A and Supplementary Table 3). In the 5th month, the *Ruminococcaceae UCG-010* (16.91%) was the predominant, followed by the genera *Ruminococcaceae UCG-005* (9.84%), *Rikenellaceae RC9 gut group* (9.37%), and *Bacteroides* (5.53%), which altogether made up 41.65% of the bacterial composition (Figure 2C and Supplementary Table 4). In the 18th month, the *Bacteroides* (10.04%) were predominant, followed by the genera *Alistipes* (9.62%), *Streptococcus* (7.49%), *Ruminococcaceae UCG-010* (6.20%), *Lactobacillus* (5.58%), and *[Eubacterium] coprostanoligenes group* (4.60%), all accounting for up to 33.49% of the bacterial community. In the 36th month, the *Bacteroides* (9.25%) was the most abundant, followed by the genera *Alistipes* (8.55%), *Streptococcus* (7.46%), *Lactobacillus* (5.48%), and *[Eubacterium] coprostanoligenes group* (5.34%), all of them accounting for 36.08% of the bacterial taxa.

### Discrepant bacterial communities in the cecum and colon microbiota of Mongolian cattle at the 5th, 18th, and 36th months of age

**FIGURE 3:**
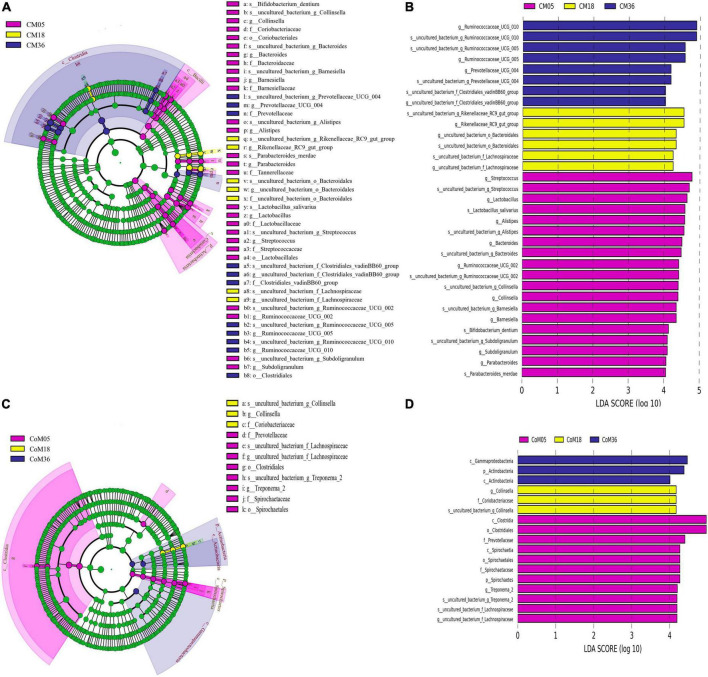
Linear discriminant effect size (LEfSe) analysis and linear discriminant analysis (LDA) of cecal and colonic microbial. Only taxa meeting an LDA threshold >4 and *p*-value threshold < 0.05 are shown in **(A)** (LEfSe) and **(B)** (LDA) for cecum and in **(C)** (LEfSe) and **(D)** (LDA) for the colon.

Linear discriminant analysis effect size (LEfSe) was used to identify microbes in the cecum of different ages after weaning (Figures 3A,B). The results showed that there were differences among the 16 genera of cecal microbiota in different months ([LDA] > 4, *P* < 0.05). *Streptococcus*, *Lactobacillus*, *Alistipes*, *Bacteroides*, *Ruminococcaceae UCG-002*, *Collinsella*, *Barnesiella*, *Subdoligranulum*, and *Parabacteroides* were highly represented in the cecum at the 5th month. In the 18th month, ASVs belonging to the *Rikenellaceae RC9 gut group*, Lachnospiraceae *uncultured bacterium*, and Bacteroidales *uncultured bacterium* were significantly enriched. *Prevotellaceae UCG 004*, *Ruminococcaceae UCG-010*, *Ruminococcaceae UCG-005*, and Clostridiales vadinBB60 group *uncultured bacterium* were highly represented in the cecum at the 36th month. In the colon, the LEfSe analysis identified statistically significant biomarkers among the groups, which were species with significant differences between groups ([LDA] > 4, *P* < 0.05). *Treponema 2* and *uncultured bacterium* were highly represented in the colon at the 5th month. *Collinsella* were highly represented in the colon at the 18th month (Figures 3C,D).

### The cecum and colon metabolome of Mongolian cattle

**FIGURE 4:**
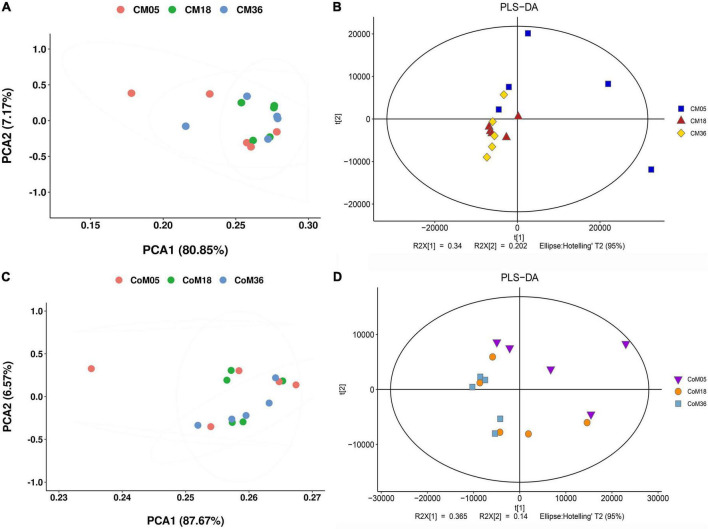
The cecum and colon metabolome of Mongolian cattle at the 5th, 18th, and 36th months of age. Principal coordinate analysis (PCA) and partial least-squares discriminant analysis (PLS-DA) reveal the metabolites variation in the cecum **(A,B)** and colon **(C,D)**, respectively.

**FIGURE 5:**
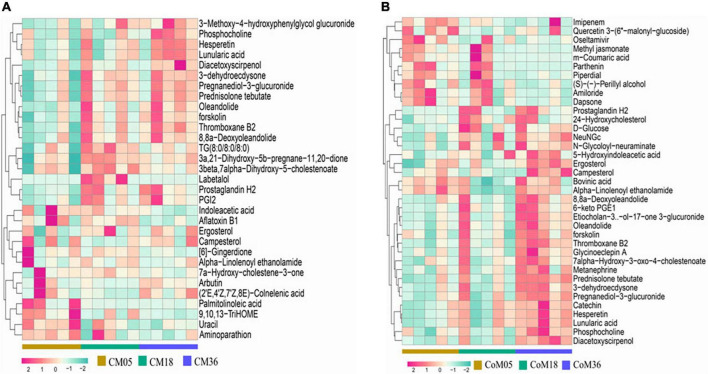
Heat map of metabolites in the cecum **(A)** and colon **(B)** of Mongolian cattle in different age groups. Relative percent abundances (square root rescaled) of top 30 metabolites in cecum (*n* = 15) and colon (*n* = 15) samples.

A total of 9,261 reliable compounds were identified from the cecum and colon samples PCA showed that metabolites in the cecum (Figure 4A) and colon (Figure 4B) did not differ among groups. The score plot of PLS-DA was performed to examine the differences or similarities in metabolites between the three age groups. The results showed differences in metabolites in the cecum (Figure 4C) and colon (Figure 4D) between the 5th-month group and the other two groups in all three age groups. In the cecum, the amounts of fatty acids such as (2′E, 4′Z, 7′Z, 8E)-colnelenic acid, palmitolinoleic acid, and 9,10, 13-trihome decreased from the 5th month to the 36th month. The contents of steroids and steroid derivatives such as pregnanediol-3-glucuronide, prednisolone tebutate, and 3a,21-dihydroxy-5b-pregnane-11,20-dione significantly increased but the content of 7a-hydroxy-cholestene-3-one decreased from the 5th month to the 36th month. The other lipid-like molecules such as lunularic acid, diacetoxyscirpenol, 3-dehydroecdysone, 8,8 a-deoxyoleandolide, TG (8:0/8:0/8:0), prostaglandin H2, PGI2, 3beta, 7alpha dihydroxy-5-cholestenoate, and oleandolide significantly increased with age (Figure 5A). The concentration of total VFAs was reduced in both colon and cecum of Mongolian cattle from the 5th month to the 36th month (*P* < 0.05) (Table 1). The contents of acetic acid, propionic acid, butyric acid, valeric acid, and isovaleric acid in the cecum decreased significantly from the 5th month to the 18th month (*P* < 0.05).

**TABLE 1:** The VFA concentrations (mmol/L) in the cecum and colon of Mongolian cattle in the different age groups.

Items	Age	ACE	PRO	IBUT	BUT	IVAL	VAL	TVFA
Cecum	5 month	11.04^a^	1.97^a^	0.15	0.82^a^	0.15^a^	0.17^a^	14.32
	18 month	5.14^b^	1.15^b^	0.11	0.28^b^	0.11^b^	0.11^b^	6.89
	36 month	6.30^b^	1.42^b^	0.14	0.38^b^	0.14^b^	0.13^b^	8.52
Colon	5 month	7.12	1.26	0.13	0.61	0.12	0.13	9.37
	18 month	6.48	1.43	0.15	0.43	0.14	0.14	8.78
	36 month	4.49	1.14	0.13	0.33	0.13	0.11	6.32

Different lowercase letters above columns indicate significant differences among different groups (P < 0.05). ACE, acetic acid; PRO, propionic acid; IBUT, isobutyric acid; BUT, butyric acid; IVAL, isovaleric acid; VAL, valeric acid; TVFA, total volatile fatty acids.

For metabolites in the colon (Figure 5B), the amounts of carbohydrates and carbohydrate conjugates such as dapsone, NeuNGc, and campesterol increase from the 5th month to the 36th month. The amounts of D-glucose, N-glycoloyl-neuraminate, 7alpha-hydroxy-3-oxo-4-cholestenoate, 8,8a-deoxyoleandolide, 6-keto PGE1, oleandolide, etiocholan-3α-ol-17-one 3-glucuronide, 24-hydroxycholesterol, catechin, phosphocholine, metanephrine, thromboxane B2, pregnanediol-3-glucuronide, 5-hydroxyindoleacetic acid, and ergosterol were higher at the 5th and 18th months than at the 36th month. In contrast, the amounts of methyl jasmonate, m-coumaric acid, quercetin 3-(6′′-malonyl-glucoside), and bovinic acid were lower in the 5th month than in the 18th and 36th months.

### Metabolic pathway analysis of metabolome data

**FIGURE 6:**
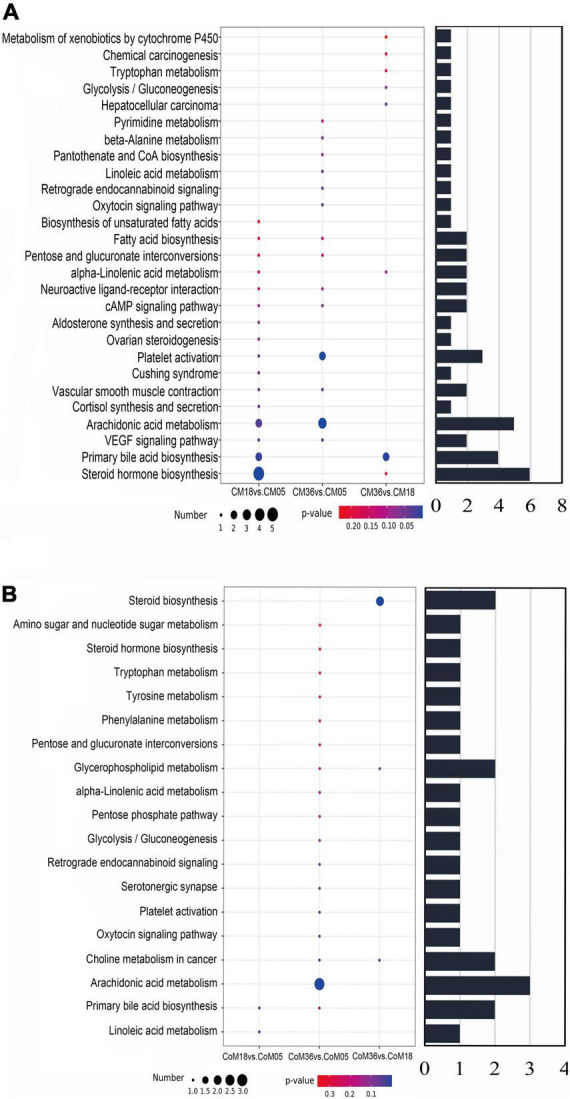
The bubble diagrams show differential metabolites in cecum **(A)** and colon **(B)** to be enriched in the pathways by KEGG analysis. The diameter of the circle shows the number of differential metabolites enriched in this pathway and the bar chart shows the total amount.

Through topology analysis by MetaboAnalyst, 27 and 19 metabolic pathways were identified to be enriched in the cecum (Figure 6A) and colon (Figure 6B), respectively. Considering both p-values (*P* < 0.05) and pathway impact values (*P* > 0.05), the differential metabolites in the cecum associated with age were mainly enriched in platelet activation (bta04611), arachidonic acid metabolism (bta00590), steroid hormone biosynthesis (bta00140), and primary bile acid biosynthesis (bta00120). While, the differential metabolites in the colon were mainly enriched in arachidonic acid metabolism (bta00590), primary bile acid biosynthesis (bta00120), choline metabolism in cancer (bta05231), steroid hormone biosynthesis (bta00140), and glycerophospholipid metabolism (bta00564).

### Correlation between bacterial community and metabolites

**FIGURE 7:**
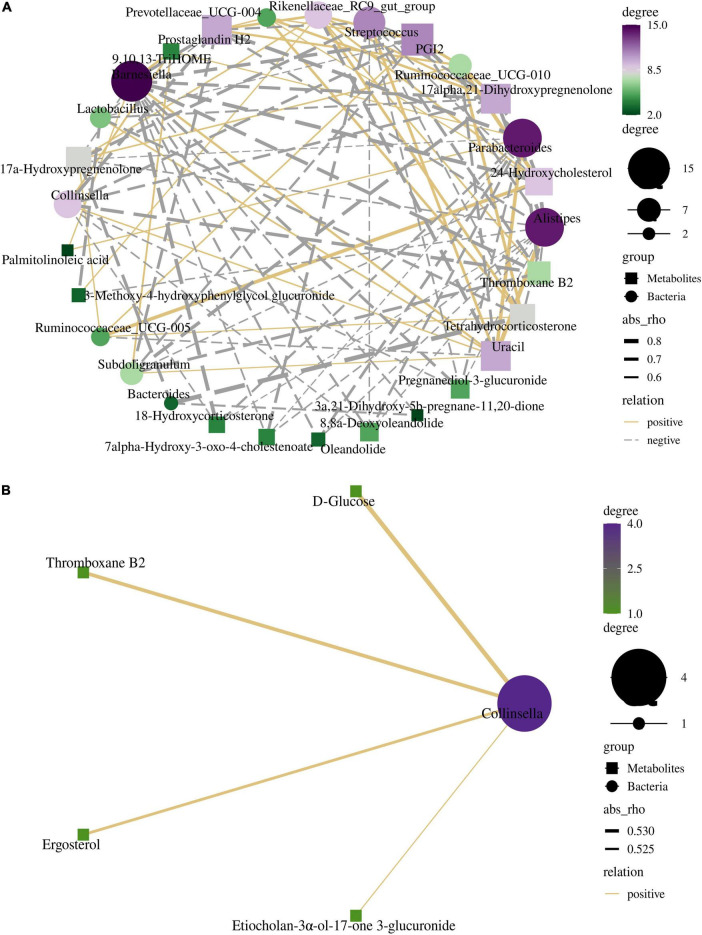
The interactive network diagrams show the correlations between the bacteria and their metabolites in the cecum **(A)** and colon **(B)**. The size of each node is proportional to the number of related metabolites or microbiome. Circular nodes represent bacteria and diamond nodes represent metabolites. The width of the line indicates the strength of the correlations. The lines in yellow and gray denote positive and negative correlations, respectively.

Spearman’s correlation analysis indicates some correlations between the relative abundances of differential bacteria and differential metabolites in the cecum and colon. The correlation networks in Figures 7A,B show the correlation between bacteria and metabolite types in the cecum and colon, respectively (details of each category are shown in Supplementary Table 5).

The relationship between differential microorganisms and metabolites in the cecum was more complicated. The differential metabolites were closely associated with *Barnesiella*, *Alistipes*, and *Parabacteroides*. All three bacterial genera mentioned above are involved in lipid metabolism. PGI2, 17alpha, 21-dihydroxypregnenolone, thromboxane B2, and prostaglandin H2 were negatively correlated with *Collinsella*, *Subdoligranulum*, *Alistipes*, *Streptococcus*, *Parabacteroides*, and *Barnesiella*, while positively correlated with *Rikenellaceae RC9 gut group*. Uracil was positively correlated with *Collinsella*, *Subdoligranulum*, *Alistipes*, *Streptococcus*, *Parabacteroide*, and *Barnesiella*, while negatively correlated with *Rikenellaceae RC9 gut group* and *Ruminococcaceae UCG-010*. *Barnesiella*, *Alistipes*, and *Parabacteroides* were negatively correlated with Pregnanediol-3-glucuronide, 17a- Hydroxypregnenolone, 8,8a-Deoxyoleandolide, 18-Hydroxyco rticosterone, Oleandolide, 7alpha-Hydroxy-3-oxo-4-chole stenoate, and 24-Hydroxycholesterol. The only differential microorganisms at the genera level in the colon were Collinsella and Treponema 2. Collinsella was positively correlated with D-Glucose, Thromboxane B2, Etiocholan-3α-ol-17-one 3-glucuronide, and Ergosterol. *Rikenellaceae RC9 gut group* was negatively correlated with the acetate acid (Supplementary Figures 4, 5). *Barnesiella* was negatively correlated with butyric acid and isobutyric acid. The ratio of acetic acid showed a significantly positive correlation with *Collinsella* and *Ruminococcaceae UCG-002*. *Ruminococcaceae UCG-002* was significantly correlated with the acetate acid. *[Eubacterium] coprostanoligens group* was negatively correlated with the butyric acid.

## Discussion

A recent study on yak rumen microbiota has shown that microbial colonization that occurs at the early stage of life may have a long-term effect on the rumen microbiota (Guo et al., 2020). The transition from breastfeeding to grass-based diets in young ruminants requires profound physiological and digestive adaptations. We studied the microbial community and metabolites in the hindgut of Mongolian cattle aged the 5th, 18th, and 36th months after weaning. Interestingly, in this study, there were significant differences in cecal bacterial diversity and richness between the 5th month and 18th, and 36th months, while there were no significant differences in colonic bacterial diversity and richness between the three age groups. The cecum of ruminants is a fermentation site other than the rumen in the digestive tract. After re-fermentation and absorption in the cecum, the nutrients in the chyme that reach the colon are more specific and homogeneous (Li S. et al., 2012; Lei et al., 2018; Zou et al., 2020). The dietary structure may be an important factor affecting the 5th-month cecal microbial diversity and richness. With the gradual stabilization of the Mongolian cattle’s diet structure, the rumen volume and fiber degradation ability gradually increased, and the microorganisms in the cecum were gradually stabilized at the 18th and 36th months. This result indicates that the selective pressure of bacteria in the cecum of grazing the Mongolian cattle may be driven by dietary fiber contents. Moreover, the intestine contains diverse microbial niches with compartmentalized physiological variations, which might be responsible for the segmented distribution of intestinal microorganisms (Li et al., 2020). Previous studies have shown that correlations between bacteria and metabolites can reflect gut maturation and mucosal growth (Slezak et al., 2013). There were more significant and complex correlations between cecal microbiota and metabolites, suggesting that the synergistic effect of cecal microbiota and metabolites improves host intestinal function.

In this study, the genera *Bacteroides*, *Streptococcus*, *Alistipes*, and *Lactobacillus* were most common in the cecum in the 5th month. These genera were also found to be abundant in the colon (Castro et al., 2016) of pre-weaning calves (Ishaq and Wright, 2012; Dill-McFarland et al., 2017), suggesting that they might play an important role in the early growth of the host. Facultative anaerobes such as *Streptococcus* and *Lactobacillus* were early colonizers to convert the GIT to a fully anaerobic environment and allow the succession and establishment of obligate anaerobes in the GIT. *Lactobacillus* can utilize lactose or other oligosaccharides in milk as substrates to produce lactate (Burgos-Rubio et al., 2000), which can be further converted to acetate, propionate, and butyrate by *C. lactatifermentans*, thereby providing energy to the host, promoting intestinal barrier function and reducing inflammation. In this study, we found that the proportion of the *Ruminococcaceae UCG-010*, *Ruminococcaceae UCG-005*, and *Rikenellaceae RC9* gradually increased and became the predominant genera in the cecum of Mongolian cattle from the 18th month to the 36th month. Members of the Ruminococcaceae are important for fiber plants degradation as demonstrated by metagenomics (Kim et al., 2011) and transcriptome analyses (Christopherson et al., 2014), and these species can contribute to the degradation of several types of polysaccharides, including starch, cellulose, and lignin in the hindgut (Chassard and Bernalier-Donadille, 2006; Flint et al., 2008; Liu et al., 2016).

Interestingly, we found an increase in the relative abundance of *Alistipes* and *Bacteroides* in the colon of Mongolian cattle from the 18th month to the 36th month. Early studies have shown that *Alistipes* contains a variety of bacteria positively related to healthy anaerobe in the host (Franzosa et al., 2019). Some *Bacteroides* have immunomodulatory activity, including *B. thetaiotaomicron* and *B. Fragilis*, which secrete polysaccharides that direct cellular and physical maturation in the development of the immune system (Mazmanian et al., 2005). Colonization of *Bacteroides* in germ-free mice was reported to correct many immune deficiencies in the gut (Ivanov et al., 2008). Our study found that *Barnesiella*, *Alistipes*, and *Parabacteroides* in the cecum are more closely related to lipids and lipid molecules, which has also been confirmed by a large number of past studies to regulate lipid metabolism (Fabersani et al., 2021; Li et al., 2022; Zhang et al., 2022). Butyrate-producing *Collinsella* provides energy to the intestinal epithelium and modulates immune function to ensure host health (Cascone et al., 2021). The results of this study showed that Ergosterol and thromboxane B2, which have bacteriostatic and antitumor effects, were positively correlated with *Collinsella*, further indicating that microorganisms play an important role in improving intestinal function. The cecum of ruminants is the second-largest fermentation site after the rumen (Li S. et al., 2012; Lei et al., 2018; Zou et al., 2020), while the colon microbiota plays an important role in host health (Hu et al., 2020). We found a close relationship between the microbial changes in the cecum and colon and the function of the intestinal segment, suggesting that intestinal function is accomplished with a variety of microorganisms settled in the intestine.

The metabolome data revealed that the metabolic differences between cecum and colon across the three age groups mainly included fatty acids and conjugates, carbohydrates and carbohydrates conjugates, and sterols. Long-chain polyunsaturated fatty acid (LCPUFA) was obtained from their dietary essential fatty acid precursors linoleic acid and α-linolenic acid (ALA) through biosynthesis (Qian and Wang, 2020). However, recent studies have found that low doses of ALA (150 mg/kg) administration profoundly ameliorated TNBS-induced clinical manifestations of colitis in mice, while high doses of ALA (300 mg/kg) did not ameliorate colitis (Wen et al., 2019) and even aggravated the symptoms. So, reduced ALA levels in the colon might be a protective mechanism against colitis. However, this still needs to be further tested on Mongolian cattle. Consistent with the previous results, the content of thromboxane B2 and PGI2 increased with the increase of Mongolian cattle age, and these lipid metabolites might be related to arachidonic acid metabolism (Reisdorf et al., 2019; Shamsi et al., 2020). Cyclooxygenase (COX) enzyme catalyzes the conversion of arachidonic acid into prostaglandin H2, which finally produces prostaglandin and other end products under the catalysis of various enzymes (Kirkby et al., 2013). Through KEGG pathway analysis, we found that the cecal and colonic differential metabolites mainly enriched in metabolic pathways that were associated with lipid metabolism, including steroid hormone biosynthesis, arachidonic acid (ARA) metabolism, and primary bile acid biosynthesis. The pathway of arachidonic acid metabolism (Oxenkrug, 2011; Yang and Liu, 2017) has immune regulatory functions in the development and manifestation of allergic diseases (Zhou et al., 2016). ARA is an essential polyunsaturated fatty acid covalently bound in the esterified form of the cellular membrane and also the direct precursor of bioactive lipid mediators, including prostaglandin, thromboxanes, and leukotrienes (Martin et al., 2016). Bile acids play a major physiological role in the solubilization and absorption of lipids and other lipophilic nutrients (Jiang et al., 2017). Generally, the bile acid signature was site-specific and most bile acids are reabsorbed by active transport in the distal ileum (Chiang, 2013). During the metabolism of bile acids, taurine- or glycine-conjugated bile acids were found to escape from the distal ileum when reabsorbed into enterohepatic circulation. However, before these bile acids escape, they are deconjugated by bile salt hydrolase secreted from gut microbiota, including *Lactobacillus* and *Bacteroides* (Wahlström et al., 2016). A small fraction of these bacterially derived bile acids are absorbed into the bloodstream and can modulate hepatic and/or systemic lipid and glucose metabolism through nuclear or G protein-coupled receptors (GPCRs) (Ghazalpour et al., 2016). Indeed, once primary bile acids reach the hindgut, they might be transformed into secondary bile acids through metabolic activities elicited by the intestinal microbial community with an accumulation of these secondary metabolites in the intestine where they can exert their anti-inflammatory properties (Alessandri et al., 2020). In conclusion, we speculated that multiple metabolic pathways (e.g., arachidonic acid metabolism and primary bile acid biosynthesis) in the hindgut of Mongolian cattle may contribute to the adaptation of Mongolian cattle to harsh environments.

VFAs are mainly derived from the metabolism of a carbohydrate and fiber-rich diet in the gut. VFAs are not only the main energy source for the host but also play important roles in regulating a variety of physiological activities (allergies and metabolic diseases) (Shortt et al., 2018). The correlation between VFA and microorganisms in the cecum was stronger than that in the colon, which was similar to previous reports that most of the chyme not fully fermented in the rumen was fermented in the cecum and other parts of the hindgut to produce final metabolites, such as volatile fatty acids (Chang et al., 2019). In this study, microbes associated with VFA were present in the cecum, such as *Streptococcus* was negatively correlated with acetic acid while *the Rikenellaceae RC9 gut group* was positively correlated with acetic acid. Consistent with previous research results, *Streptococcus* is the main acetic acid-producing microorganism in the intestinal tract (Duttaroy, 2021). Surprisingly, the VFAs (acetic, propionic, butyric, isovaleric, and valeric) in the cecum decreased with age. After weaning, with the increase in feed intake, rumen function gradually improved, and more nutrients and structural polysaccharides were degraded and absorbed in the rumen (Dijkstra, 1994). There was no significant difference in the content of volatile fatty acids in the colon, probably because the volatile fatty acids in the chyme were digested before the colon (Shannon et al., 2021).

## Conclusion

This study comprehensively explored the post-weaning hindgut microbiota and metabolome in grazing Mongolian cattle for the first time. The results of the studies on the diversity and metabolites of hindgut microbiota suggested that the diversity of cecum microbiota increased with age, and the perfection of colon microbiota might be completed before weaning. Compared with the 5th month of age, the dominant bacterial community in the cecum at the 18th and 36th months of age shifted toward microbes associated with the fermentation and degradation of plant-derived cellulose, while the immune-related microbiota in the colon gradually increased. Microorganisms and metabolites in the cecum and colon and their interactions together increase the adaptability of Mongolian cattle in harsh environments.

## Data availability statement

The datasets presented in this study can be found in online repositories. The names of the repository/repositories and accession number(s) can be found below: NCBI BioProject–PRJNA843569.

## Ethics statement

All procedures involving animals were approved by the Animal Administration and Ethics Committee of Lanzhou Institute of Husbandry and Pharmaceutical Sciences, Chinese Academy of Agricultural Sciences, Lanzhou, China (2019-0004).

## Author contributions

ZL, JiZ, and MD collected the samples. BT, ZL, JuZ, and JiZ prepared the samples for analysis. AA analyzed the data. ZL wrote the manuscript. SW and GS investigated. PY supervised. JH, BT, and XD reviewed the manuscript and project administration. All authors approved the final manuscript as submitted, contributions to the research, and agreed to the published version of the manuscript.

## Conflict of interest

The authors declare that the research was conducted in the absence of any commercial or financial relationships that could be construed as a potential conflict of interest.

## Publisher’s note

All claims expressed in this article are solely those of the authors and do not necessarily represent those of their affiliated organizations, or those of the publisher, the editors and the reviewers. Any product that may be evaluated in this article, or claim that may be made by its manufacturer, is not guaranteed or endorsed by the publisher.

## Footnotes

1
http://www.hmdb.ca/

2
http://www.lipidmaps.org/

3
http://www.cytoscape.org

4
https://www.r-project.org
