# Supplementary material for: Age-dependent changes of hindgut microbiota succession and metabolic function of Mongolian cattle in the semi-arid rangelands
Source: Front Microbiol. 2022 Jul 22;13:957341. doi: 10.3389/fmicb.2022.957341 (PMC9354825; doi:10.3389/fmicb.2022.957341)
Supplement: Supplementary file 2 [file Table_2.DOCX]

**Supplementary information**

**
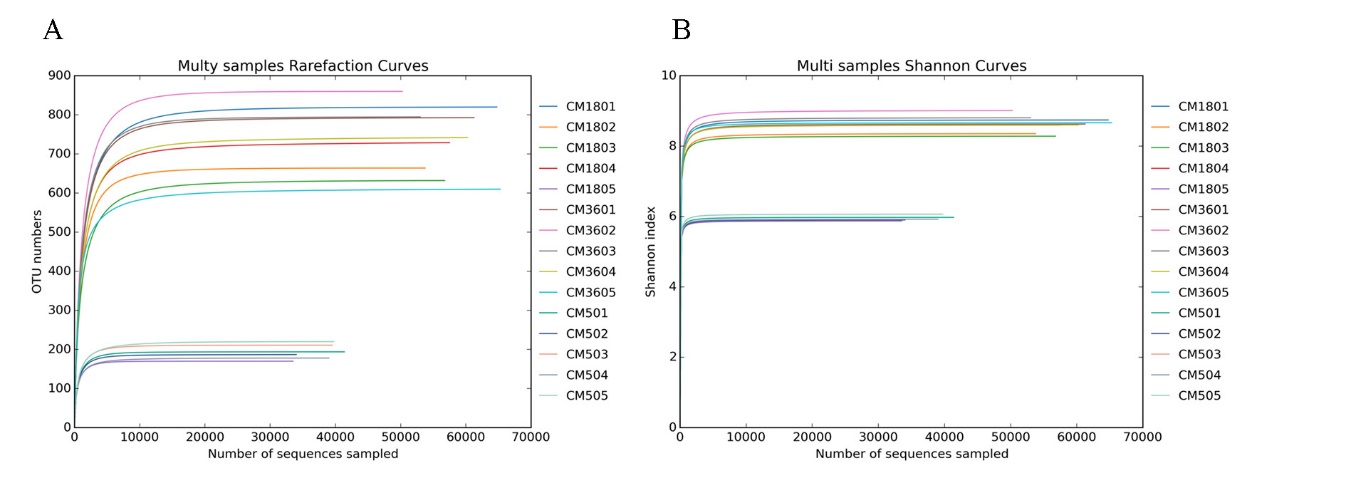
**

**Supplementary Fig. S1** Rarefaction curves (A) and Shannon curves (B) of bacteria in cecal contents of Mongolian cattle.

**
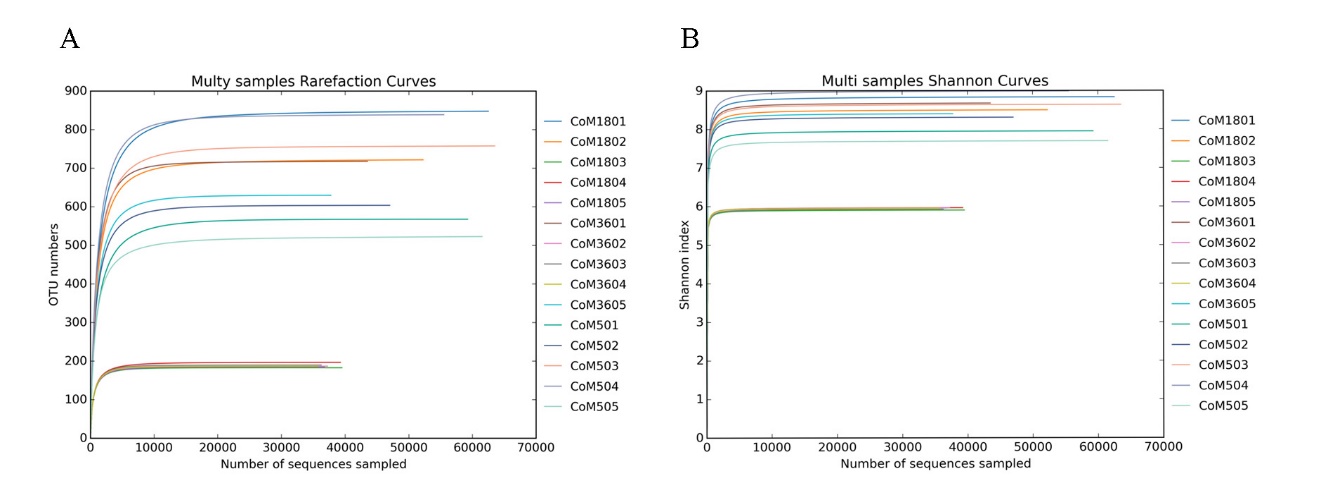
**

**Supplementary Fig. S2** Rarefaction curves (A) and Shannon curves (B) of bacteria in colonic contents in Mongolian cattle.

**
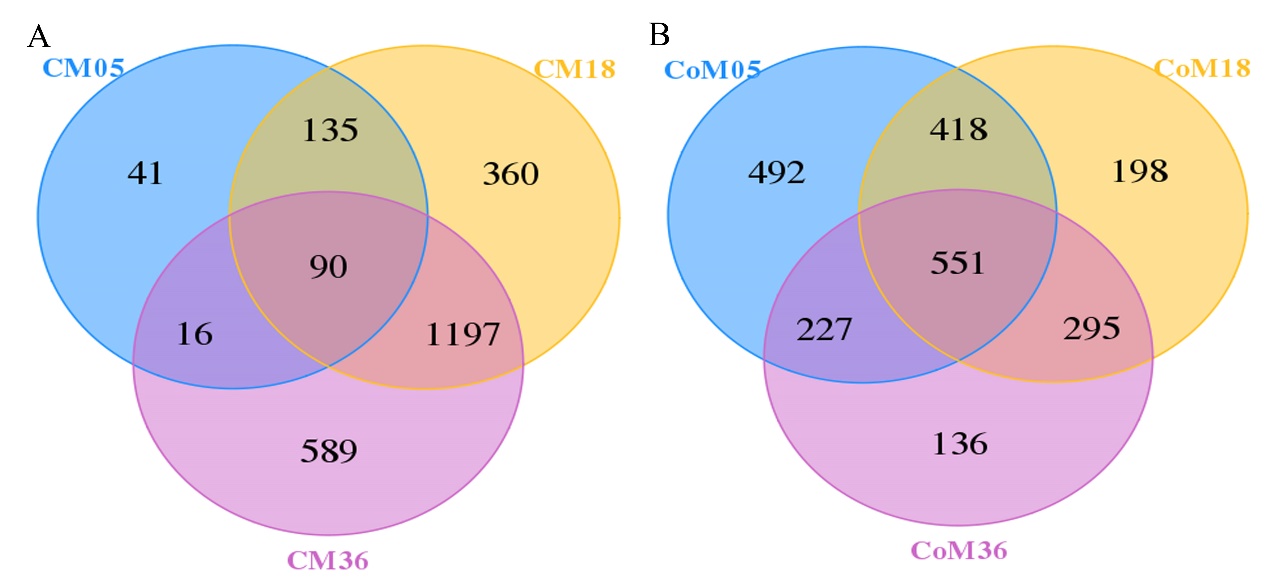
**

**Supplementary Fig. S3** Venn diagram of bacterial ASVs in cecum and colon of Mongolian cattle after weaning. Cecum (A) and colon (B) samples are displayed separately.

**
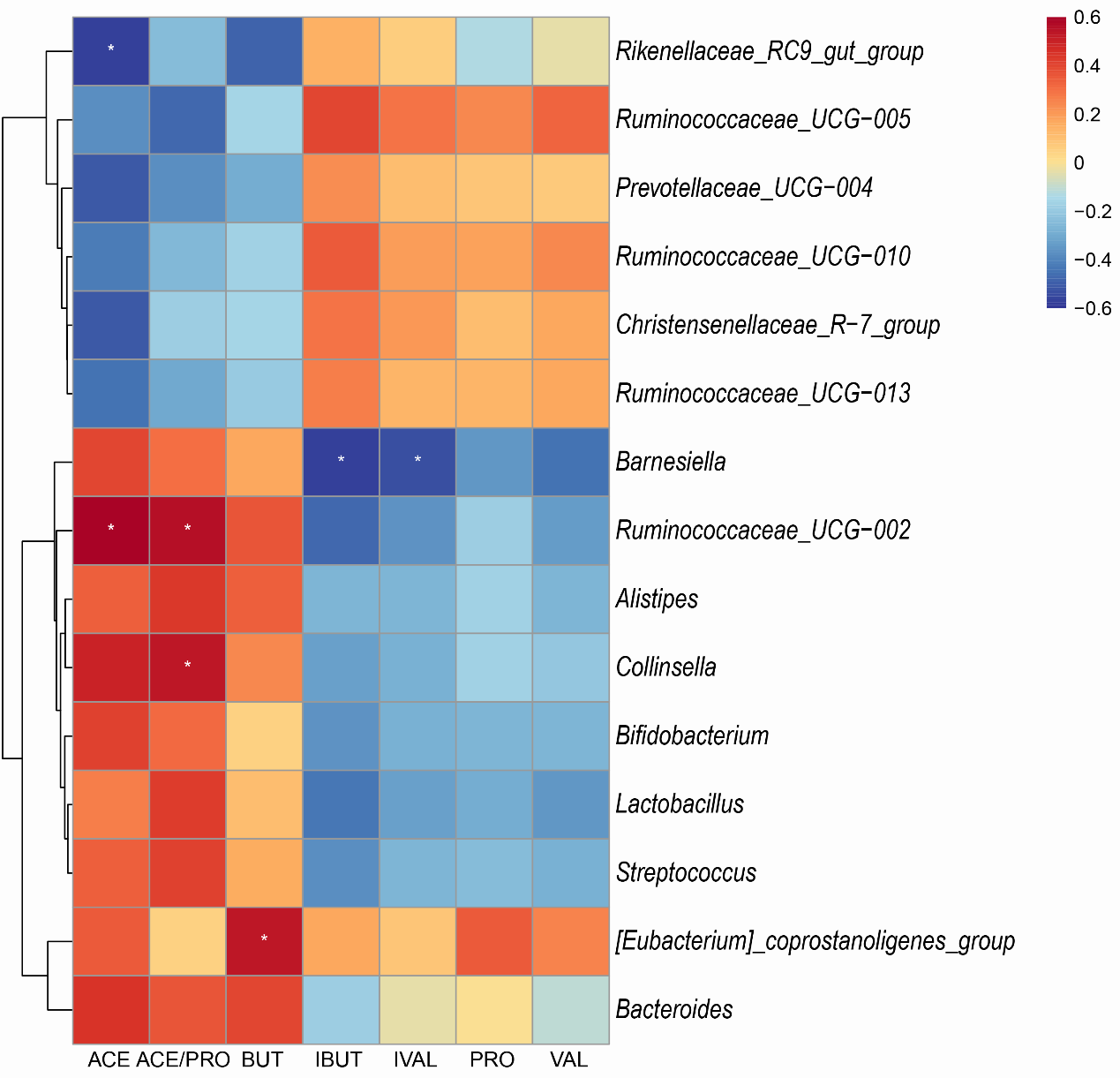
**

**Supplementary Fig. S4** Heatmap of the correlation between volatile fatty acids and bacteria in the cecum. Correlation coefficient greater than 0.5 indicates strong correlation, which is denoted as "*".

**
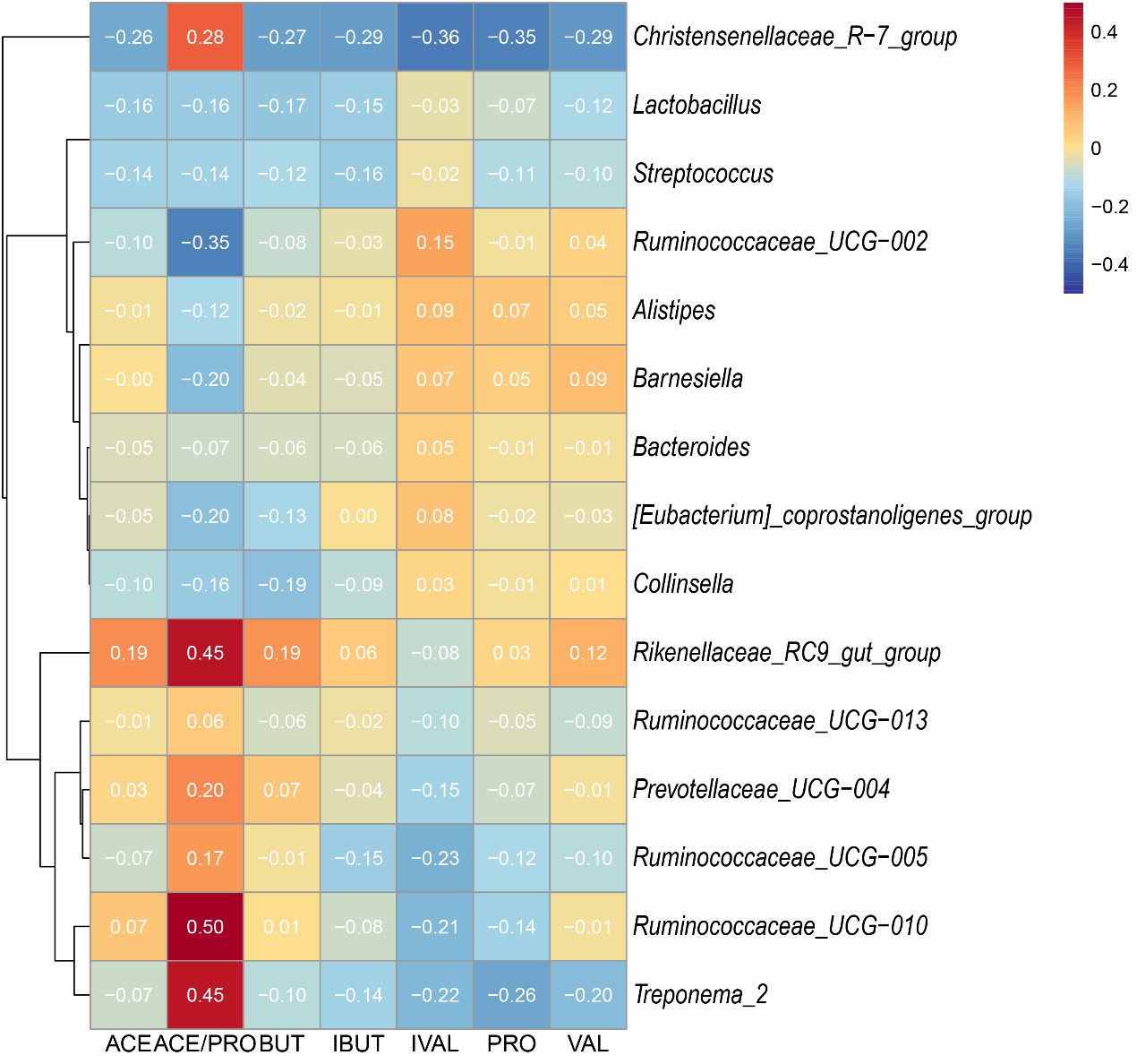
**

**Supplementary Fig. S5** Heatmap of the correlation between volatile fatty acids and bacteria in the colon.
